# Towards a Long-Term Strategy for Voluntary-Based Internal Radiation Contamination Monitoring: A Population-Level Analysis of Monitoring Prevalence and Factors Associated with Monitoring Participation Behavior in Fukushima, Japan

**DOI:** 10.3390/ijerph14040397

**Published:** 2017-04-09

**Authors:** Shuhei Nomura, Masaharu Tsubokura, Akihiko Ozaki, Michio Murakami, Susan Hodgson, Marta Blangiardo, Yoshitaka Nishikawa, Tomohiro Morita, Tomoyoshi Oikawa

**Affiliations:** 1 Department of Epidemiology and Biostatistics, School of Public Health, Imperial College London, Norfolk Place, London W2 1PG, UK; 2 Department of Global Health Policy, Graduate School of Medicine, The University of Tokyo, 7-3-1 Hongo, Bunkyo-ku, Tokyo 113-0033, Japan; 3 Department of Radiation Protection, Minamisoma Municipal General Hospital, 2-54-6 Takami-cho, Haramachi-ku, Minamisoma, Fukushima 975-0033, Japan; tsubokura-tky@umin.ac.jp (M.T.); minamisoma-kyukyu@city.minamisoma.lg.jp (T.O.); 4 Department of Surgery, Minamisoma Municipal General Hospital, 2-54-6 Takami-cho, Haramachi-ku, Minamisoma, Fukushima 975-0033, Japan; ozakiakihiko@gmail.com; 5 Department of Health Risk Communication, Fukushima Medical University School of Medicine, 1 Hikarigaoka, Fukushima, Fukushima 960-1295, Japan; michio@fmu.ac.jp; 6 Radiation Medical Science Center for the Fukushima Health Management Survey, Fukushima Medical University, 1 Hikarigaoka, Fukushima, Fukushima 960-1295, Japan; 7 MRC-PHE Centre for Environment and Health, Department of Epidemiology and Biostatistics, School of Public Health, Imperial College London, Norfolk Place, London W2 1PG, UK; susan.hodgson@imperial.ac.uk (S.H.); m.blangiardo@imperial.ac.uk (M.B.); 8 Department of Health Informatics, School of Public Health, Kyoto University, Yoshida-Konoe, Sakyo-ku, Kyoto 606-8501, Japan; ynishikawa-tky@umin.ac.jp; 9 Department of Radiation Protection, Soma Central Hospital, 3-5-18 Okinouchi, Soma, Fukushima 976-0016, Japan; t.morita526@gmail.com

**Keywords:** Japan’s 2011 Fukushima nuclear incident, voluntary internal radiation monitoring program, monitoring behavior

## Abstract

Following Japan’s 2011 Fukushima nuclear incident, we assessed voluntary-based monitoring behavior in Minamisoma City—located 10–40 km from the Fukushima nuclear plant—to inform future monitoring strategies. The monitoring in Minamisoma included occasional free of charge internal-radiation-exposure measurements. Out of around 70,000 individuals residing in the city before the incident, a total of 45,788 residents (female: 52.1%) aged ≥21 were evaluated. The monitoring prevalence in 2011–2012 was only 30.2%, and this decreased to 17.9% in 2013–2014. Regression analyses were performed to estimate factors associated with the monitoring prevalence and participation behavior. The results show that, in comparison with the age cohort of 21–30 years, the cohort of 71–80 and ≥81 years demonstrated significantly lower monitoring prevalence; female residents had higher monitoring prevalence than male residents; those who were living in evacuation zones at the time of the incident had higher monitoring prevalence than those who lived outside any of the evacuation zones; for those living outside Fukushima and neighboring Prefectures post-incident monitoring prevalence decreased significantly in 2013–2014. Our findings inform the discussion on the concepts of radiation risk perception and accessibility to monitoring and societal decision-making regarding the maintenance of the monitoring program with low monitoring prevalence. We also stress the possibility that the monitoring can work both to check that internal contamination levels are within acceptable limits, and as a risk communication tool, alleviating individuals’ concern and anxiety over radiation contamination.

## 1. Introduction

Radiation exposure is a public health issue, associated with acute radiation syndrome, gastrointestinal and/or hematologic morbidity and mortality, and long-term risks of disorders including cancer [1]. After Japan’s 2011 Fukushima Daiichi Nuclear Power Plant incident, that followed the Great East Japan Earthquake on 11 March 2011, anxiety over the incident has arisen in the general public [2] and external and internal radiation exposure is the major public concern in radiation-contaminated areas [3]. 

Radiation dose from external radiation exposure highly depends on air dose rates at places where individuals spend long periods of time; lifestyle habits have limited impacts on external dose [4]. On the other hand, dose from internal exposure is strongly influenced by modifiable dietary habits, including consumption of contaminated local food products [5]. Owing to the quick response by the Japanese central government and local authorities [6], including foodstuff contamination management via the radiation inspection of foodstuffs circulating on the market [7,8] and monitoring of internal contamination levels using whole body counter (WBC) units and identification of risk behavior [9,10], internal dose among the majority of the population in the contaminated areas is already marginal and mostly undetectable by WBC [9,11]. As a result, Tsubokura et al. acknowledged that a large proportion of individual’s cumulative dose can be attributed to external radiation exposure [12].

To maintain low levels of internal radiation contamination among residents in radiation-contaminated areas, an internal contamination monitoring particularly plays an important role [13]. In both emergency phase (for which immediate decisions for effective use of protective actions are required) and intermediate phase (after the source and releases of radioactive effluents have been brought under control until additional protective actions are no longer needed), monitoring strategies for internal contamination in the affected areas should specify clear objectives, such as: identifying people with high levels of internal contamination relative to the population average; assessing internal dose at the population level; and informing personal and public health measures (e.g., urgent decontamination, counseling and dietary advice) [13].

Importantly, the long-lived Cs (with a physical half-life of 30 years for ^137^ Cs—the major constituent of the aerial release after a nuclear incident) may pose a persisting long-term risk of internal exposure via intake of contaminated products. After the 1986 Chernobyl nuclear incident, internal exposure contributed substantially to the cumulative, long-term radiation exposure in the general public, which was largely due to intake of locally grown produce with sustained radio-contamination [14]. In the case of the Fukushima nuclear incident, five years after the incident, although the cumulative dose is predominantly due to external radiation exposure [12], certain residents in the affected areas still sometimes show relatively high (compared to the average levels) or detectable levels of internal contamination [15], also due to radio-contamination of locally grown uninspected produce. Levels of internal contamination in these cases have occasionally reached over 100 Bq/kg of radiocesium (Cs), comparable to levels observed after the Chernobyl incident [16]. Therefore, an internal monitoring program might be also beneficial in a post-immediate phase (i.e., recovery phase)—the period until when all recovery actions initiated to reduce radiation levels in the environment to acceptable levels have been completed [17,18]. However, there is a little knowledge on how to better design a long-term monitoring strategy, including the purposes and objectives of monitoring, who should be monitored, over which time periods, etc.

Receipt of public services, including medical assessment, counseling and treatment should be optional under the banner of liberalism and democracy (aside from the cases where people, if untreated or undiagnosed, represent a danger to themselves or others). In any phase of a nuclear disaster (from emergency to recovery phase), great caution is requisite in the development of the ‘default settings’ for the exposure monitoring, including whether monitoring participation is mandatory or voluntary. The choice of the default setting can lead (i.e., nudge [19]) people to create/increase or moderate their anxieties and concerns over radiation exposure [20,21], determining overall public health harms/benefits of monitoring. After the Fukushima incident, the central and local authorities in the affected areas decided that voluntary participation was the preferred option for people in the affected areas, which may well agree with the philosophy of libertarian paternalism—a concept derived from cognitive psychology and behavioral science, which aims to encourage individuals to make choices which are in their best interests, while maintaining their freedom of choice [19,22]. 

We have been supporting clinical care and research in Minamisoma City, Fukushima Prefecture, Japan, located 10–40 km from the Fukushima nuclear power plant, with a pre-incident population of about 70,000. In response to radiation concerns among the city residents, Minamisoma City led the vanguard among the affected municipalities by launching the first voluntary internal contamination monitoring program for a population in the city, four months following the incident (July 2011). Five years after the incident, marginal internal contamination is evident [9,11], and Minamisoma City (as well as other concerned local and public health authorities and professionals in the affected areas) are now standing at a crossroad having to decide how to design the internal contamination monitoring strategy in a long-term perspective. In this regard, a comprehensive understanding of monitoring behavior among the population will enhance discussions regarding societal decision-making on the continued operation of the present monitoring program. 

This study, therefore, aimed to understand monitoring participation behavior of residents in Minamisoma City, and the factors associated with this behavior. With the cooperation of the Minamisoma City Office and the two WBC-installed hospitals in the city, we addressed the following three objectives: (1) to estimate the WBC monitoring prevalence (coverage) in the city and the change in prevalence over time; (2) to identify factors associated with the monitoring prevalence; and (3) to identify characteristics of individuals that influence their monitoring participation behavior over time.

## 2. Materials and Methods 

### 2.1. Setting 

Minamisoma City (Fukushima Prefecture, Japan), is located 10–40 km from the Fukushima nuclear power plant, and the pre-incident population (as of February 2011) of Minamisoma City was 71,494 [23]. On 12 March 2011, a 20 km radius from the Fukushima nuclear plant was denoted by the central government as a restricted area with compulsory evacuation, and residents within a 20–30 km radius were ordered on 15 March to shelter in place [24]. Minamisoma City therefore straddles the initial compulsory evacuation zone (where about 17 thousand people had lived, representing 24.7% of the total population of Minamisoma City) and indoor-sheltering zone [23]. 

**Figure 1:**
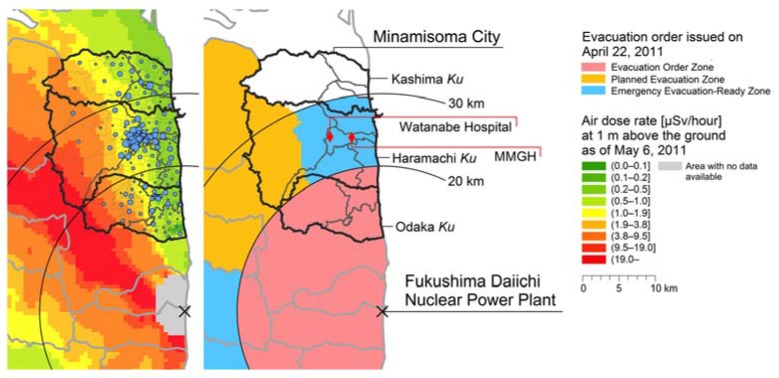
Locations of Minamisoma City and two whole body counter-installed hospitals. The base map shows the air dose rate [μSv/h] as of 22 April 2011 at a height of 1 m above the ground measured in terms of the ambient dose equivalent (H*10) [25], which includes the natural radiation background from the earth’s crust. Data source details are explained in the methods section of this paper. The blue circles show the geographical distribution of the study population, where the circles are proportional to the number of subjects living in each Oaza. MMGH indicates Minamisoma Municipal General Hospital.

On 22 April 2011, the compulsory evacuation zone was expanded slightly to the northwest based on the measured dispersion of highly radioactive fallout (Figure 1), and the evacuation/indoor-sheltering zones were reclassified into three zones in line with air dose rates: (a) Evacuation Order Zone; (b) Planned Evacuation Zone; and (c) Emergency Evacuation-Ready Zone. On 12 July 2016, the central government lifted the evacuation order for all but a tiny slice of the city. The geographical scope of these zones and the locations of Minamisoma City and the two WBC installed hospitals (see below section), relative to the nuclear power plant, are shown in Figure 1.

### 2.2. Data Collection

To meet our objectives, we collected data from the ‘Whole Body Counter database’ and ‘evacuation database’ of Minamisoma City, described below.

#### 2.2.1. Whole Body Counter Database

In response to the Fukushima incident, Minamisoma City launched a voluntary internal radiation exposure monitoring program for the city residents in July 2011, using WBC units. The monitoring was initially performed using a chair-type WBC (Anzai Medical Co., Ltd., Tokyo, Japan), installed in a bus-like vehicle located in the parking lot of Minamisoma Municipal General Hospital (MMGH). From August 2011, a second chair-type WBC (Fuji Electric Co., Ltd., Tokyo, Japan) was installed at MMGH. However, because these WBCs provided insufficient shielding against background gamma rays [15], they were replaced by a better-shielded standing-type WBC (FASTSCAN Model 2251, Canberra Inc., Meriden, CT, USA) on September 2011. From July 2012, another chair-type WBC unit (WBC-R43-22458, Hitachi Aloka Medical, Ltd., Tokyo, Japan) was installed at Watanabe Hospital, located approximately 3 km to the west of MMGH. More detailed product information can be found elsewhere [15,26]. Locations of these hospitals are shown in Figure 1. Notification of the monitoring program was disseminated using the city’s official website and public magazine, monitoring for the Minamisoma residents is free of charge (except for travel expenses), by appointment only.

We obtained the WBC records for each participating resident from the inception of the monitoring program to 31 March 2015. These data comprise: gender, age at 11 March 2011 (classified in ten-year intervals: 0–10, 11–20, 21–30, 31–40, 41–50, 51–60, 61–70, 71–80, and ≥81), person identification number, date of measurement, and results of the measurement (i.e., body burden [Bq/body] of ^134^ Cs and ^137^ Cs).

#### 2.2.2. Definition of the ‘WBC Monitoring Prevalence’

When the WBC monitoring started in July 2011, appointments were booked more than a half year in advance. Because of the large number of pre-booked appointments, residents could not usually participate in the monitoring more than once until March 2013. After that time, as waiting times for appointments decreased slightly, it was possible for participants to have repeat measurements in the same year. For these reasons, the study observation period was split at March 2013 into two time periods for analyses: 1 July 2011 to 31 March 2013 (2011–2012); and 1 April 2013 to 31 March 2015 (2013–2014). Accordingly, we defined the WBC monitoring prevalence as the number of Minamisoma City residents who participated in the monitoring during each period, divided by the total number of the Minamisoma residents (population) at each period who met eligibility criteria (see below). If an individual participated more than once in each period, the data at the time of their first participation was considered for each period.

#### 2.2.3. Evacuation Database

After the Fukushima incident, Minamisoma City experienced a large population movement resulting from evacuations. Proper estimates of the WBC monitoring prevalence require accurate data of post-incident population demographics of the city residents; it was therefore indispensable to know accurate dwelling address of the city residents post-incident—i.e., who lived where at what time point.

In Japan, there is the nationwide resident-registry network, the “Basic Resident Register”, administrated by each municipality unit (city/town/village). This register contains basic data of registered residents, such as name, gender, date of birth and address information. After the incident, because evacuees often did not change the address recorded in the Basic Resident Register of their original municipality, the registered residential address post-incident does not necessarily indicate actual lived-at address. Evacuees did, however, report their evacuation/relocation status, including dwelling address information, to the Minamisoma City Office, in order to receive important notifications from the office, such as tax payment, disaster recovery insurance, and compensation claims. Note, in this study ‘evacuees (evacuations)’ include ‘relocatees (relocations)’, defined as those who evacuated after the incident and requested a municipality office change their registered residential address. In 2015, Minamisoma City created an ‘evacuation database’ by combining an individuals’ evacuation records and their corresponding Basic Resident Register data. Although the evacuation-record reporting was not mandatory, given the legal necessity to receive tax and insurance notices, we considered that this database was complete, and contained the evacuation records of all Minamisoma residents. 

From this evacuation database, we extracted the person identification number (to enable linkage to the WBC database) and dwelling address (where people are actually living) at 1 June 2011 and 1 June 2013—to reflect residence in the two study periods. Denominator populations for the WBC monitoring prevalence for each study observation period were then based on the total number of registered residents at 1 June 2011 and 1 June 2013, respectively.

In addition, in order to know original dwelling address at the time of the incident (11 March 2011), and for the use in determining eligibility (see below), we also obtained data regarding the registered residential address, as of 1 March 2011 before the incident and 1 June 2015 (date after the study period) from the Basic Resident Register, via the person identification number. Note that address information was de-identified prior to the data collection, so we were not able to access full address, instead obtained the *Oaza* (i.e., sub-district), the lowest hierarchy of administrative divisions of Minamisoma City. Information on the city’s administrative divisions are explained in the section below.

### 2.3. Eligibility Criteria of Study Subjects

In addition to the voluntary monitoring scheme described above, Minamisoma City implements a mandatory WBC monitoring program for all primary (ages 7–12 years) and secondary (13–15 years) school children in the city, in the form of an annual school health check-up since April 2013 in order to respond to parents’ persisting concerns. Because of the nature of these different monitoring schemes, we excluded age cohorts of 0–10 and 11–20 years; so age cohorts in decades from 21–30 years to ≥81 years at the time of the incident were considered. In addition, we exclusively considered those who were registered as Minamisoma City residents both as of 1 June 2011 and 1 June 2015. By doing so, we could follow the study subjects’ WBC monitoring participation behavior during the study period.

### 2.4. Data Analysis

We performed the following three analyses: 

#### 2.4.1. Analysis 1: Relative Prevalence of WBC Monitoring in 2013–2014 vs. 2011–2012 

To estimate the change of the WBC monitoring prevalence over time, we calculated the monitoring prevalence for 2011–2012 and 2013–2014, and relative prevalence (RP) of 2013–2014 vs. 2011–2012.

#### 2.4.2. Analysis 2: Factors Associated with the WBC Monitoring Prevalence in 2011–2012 and 2013–2014

To identify demographic factors associated with the monitoring prevalence, and differences by original dwelling address and post-incident dwelling area, we performed multivariate regression analyses. Regression models were constructed separately for each time period, so that we could see how the magnitude and significance of any effects on the WBC monitoring prevalence changed over time.

##### Regression Model

Negative binomial models are an appropriate analytical technique in this analysis because they assume log linear rate function between a dependent variable (here, monitoring prevalence) and independent variables. The regression results indicate the multiplicative change in the WBC monitoring prevalence for a unit increase in each variable (i.e., prevalence ratio: PR). 

##### Variables Considered

The independent variables included age at 11 March 2011 (in ten-year intervals), gender, original residential area, and post-incident dwelling area at 1 June 2011 and 1 June 2013. 

#### 2.4.3. Analysis 3: Factors Associated with the WBC Monitoring Participation Behavior

To identify characteristics of individuals that were associated with monitoring participation behavior over time, we also constructed multivariate regression models.

##### Regression Model

We considered the ‘monitoring participation pattern’ as the dependent variable in the models, and defined four distinct ‘patterns’: (I) participated both in 2011–2012 and 2013–2014; (II) participated only in 2011–2012; (III) participated only in 2013–2014; and (IV) participated in neither 2011–2012 nor 2013–2014 (non-participation).

We used a multinomial logistic regression model with ‘non-participation’ as the reference group, which allowed for the use of such a categorical dependent variable. By the properties of the multinomial logistic model, the effect of each independent variable was computed as the multiplicative change of odds of adopting pattern I–III vs. non-participation (pattern (IV)) for a unit increase in the variable (i.e., odds ratio: OR).

##### Variables Considered

The independent variables included age as of 11 March 2011 (in ten-year intervals), gender, original residential area, and evacuation history. For this analysis, the evacuation history was divided into four categories: (a) evacuated both in 2011–2012 and 2013–2014; (b) evacuated in 2011–2012, but returned to original residential area by 2013–2014; (c) evacuated only during 2013–2014; and (d) evacuated in neither 2011–2012 nor 2013–2014 (non-evacuation). In this analysis, we defined evacuation as a movement away from the original residential *Ku*. Note that Minamisoma City is divided into a hierarchy of administrative divisions: from the highest to the lowest, *Ku* (i.e., area, n = 3), *Chiku* (i.e., district, n = 12), and *Oaza* (i.e., sub-district, n = more than 100). Figure 1 illustrates the geographical location of these three *Ku* within the city. As a sensitivity analysis, we also constructed regression models using a different definition of ‘evacuation’—a movement away from the original residential *Chiku* instead of *Ku.*

We also considered *Oaza*-level radiation air dose rates (μSv/h at a height of 1 m above the ground measured in terms of the ambient dose equivalent (H*10) [25], obtained from the official website of the Ministry of Education, Culture, Sports, Science, and Technology (MEXT)) at original residential area as an independent variable. All monitored results are open to public and available online [27]. We considered the results of the first MEXT monitoring performed between 6 April 2011 and 29 April 2011 [27]. The *Oaza*-level radiation level was then calculated by averaging the values at each monitoring point within the *Oaza* area, and this *Oaza* average value was then assigned to each individual based on original residential area at 1 March 2011.

##### Sub-Analysis for the Consideration of ‘Being Detected’ with Cs

It is reasonable to think that being detected vs. non-detected with Cs in the first WBC measurement will influence whether or not a resident would undergo a subsequent WBC measurement. Therefore, the detection of Cs would ideally be included as an independent variable in the regression analyses. However, as described earlier, chair-type WBC units used at MMGH from the inception of the WBC monitoring in July 2011 until September 2011, had an insufficient shielding against background gamma rays, which made the measurement of internal contamination as well as clinical judgment of ‘being detected’ with Cs less reliable.

Therefore, to properly evaluate the effect of the detection of Cs, we conducted sub-analyses considering only those who participated in the WBC monitoring after September 2011 up to 31 March 2013 using FASTSCAN at MMGH or WBC-R43-22458 at Watanabe Hospital, and examined factors associated with whether or not they had a subsequent WBC monitoring participation in the second period (2013–2014). Thus, the dependent variable is binary (participated in the WBC monitoring in 2013–2014 or not) and independent variables included were the same with the above analysis; the logistic regression model was applied to estimate odds ratio for participating in the WBC monitoring in 2013–2014. Note that the detection limits of the WBC units at MMGH and Watanabe Hospital are 220 Bq/body for ^134^ Cs and 250 Bq/body for ^137^ Cs following a two-minute scan. We used STATA/MP version 13.1 (StataCorp LLC, College Station, TX, USA) for all analyses, and a *p*-value of less than 0.05 was considered statistically significant.

### 2.5. Ethics Approval

Ethical approval for this study was granted by the ethics committee of the Minamisoma Municipal General Hospital (MMGH) in Minamisoma City, authorization number 28-02. The ethics committee agreed that written consent was not required for each individual because this study was performed retrospectively.

## 3. Results

### 3.1. Demographic Characteristics

The population across all eligible age cohorts who were registered as Minamisoma City residents both at 1 June 2011 and 1 June 2015, was 45,788 (female: 52.1%). Pre-incident demographic characteristics are shown in Table 1. The majority of residents (85.3%) lived in one of the three evacuation zones, with 20.7% forced to evacuate following the Fukushima incident. Because the number of those living in the Planned Evacuation Zone at the time of the incident was small (n = 9) and they were forced to evacuate before the monitoring program started (July 2011), we included these 9 residents in the Evacuation Order Zone in the analyses.

Post-incident demographic characteristics of the study population are also shown in Table 1. While many residents were living outside Minamisoma City as of 1 June 2011 (48.8%), about half of them had returned to the city two years later (25.8%). Median air dose rate [μSv/h] as of 22 April 2011 at original residential area in the study population was 0.86 with interquartile range of 0.43–1.28, including the natural radiation background from the earth (about 0.04 μSv/h).

### 3.2. Analysis 1: Relative Prevalence of WBC Monitoring in 2013–2014 vs. 2011–2012

In 2011–2012, the WBC monitoring prevalence was 30.2% overall (Table 2). Female residents were more likely to participate in the monitoring than males (female 32.9% vs. male 27.2%, *p* < 0.001: chi-squared test). The age cohort with the lowest monitoring prevalence was that of ≥81 years for both female and male (9.4% and 13.6% respectively).

Meanwhile, in 2013–2014, the WBC monitoring prevalence dramatically decreased to 17.9% overall (female: 19.6%; male: 16.0%); the RP in 2013–2014 vs. 2011–2012 was 0.59 (95% Confidence Interval (CI): 0.58–0.61). In all the age cohorts, a substantial decline in the monitoring prevalence was observed. The RP shows that the decline in monitoring prevalence, in males, was predominantly in younger age cohorts. In females, the similar age-RP tendency was observed with exceptions for the cohorts of 21–30 and ≥81 years. Further details can be found in Table 2.

### 3.3. Analysis 2: Factors Associated with the WBC Monitoring Prevalence in 2011–2012 and 2013–2014

Results of the regression analyses are shown in Table 3. In 2011–2012, in comparison with the age cohort of 21–30 years, the cohort of 71–80 and ≥81 years demonstrated significantly lower monitoring prevalence (PR: 0.86, 95% CI: 0.77–0.96, *p* < 0.01; PR: 0.35, 95% CI: 0.30–0.41, *p* < 0.001, respectively), after adjustment for the covariates. Female residents had higher monitoring prevalence than male (PR: 1.25, 95% CI: 1.18–1.32, *p* < 0.001). Those who were living in the Evacuation Order Zone or Emergency Evacuation-Ready Zone at the time of the incident had higher monitoring prevalence than those in outside any of evacuation zones (PR: 1.78, 95% CI: 1.57–2.03, *p* < 0.001; PR: 1.51, 95% CI: 1.36–1.68, *p* < 0.001, respectively). Post-incident dwelling area had no statistically significant association with WBC monitoring prevalence in 2011–2012 but for those living outside Fukushima and neighboring Prefectures, monitoring prevalence decreased significantly in 2013–2014.

In order to assess whether the effect of post-incident dwelling area on the monitoring prevalence differed by original residential area, the final model presented in Table 3 also included the interaction of the original residential area and post-incident dwelling area (Table 4). 

In 2011–2012, among those who were living outside the evacuation zones at the time of the incident, there was no significant effect of the post-incident dwelling area on the monitoring prevalence. However, for those who were living in the Evacuation Order Zone or Emergency Evacuation-Ready Zone at the time of the incident, the post-incident dwelling area had a significant effect on the monitoring prevalence. Those living outside of Minamisoma City had lower monitoring prevalence than those living inside the city. Similar results were observed in 2013–2014.

### 3.4. Analysis 3: Factors Associated with the WBC Monitoring Behavior

Summary statistics of the WBC monitoring participation patterns are shown in Table 5. Percentage of those who participated in the WBC monitoring both in 2011–2012 and 2013–2014 (pattern (I)) was 9.7% overall (female: 10.8%; male: 8.5%). Both in females and males, the age cohort with the highest proportion of pattern (I) was 61–70 years (female: 14.8%; male: 12.6%), while the cohort of ≥81 years had the lowest and 2nd lowest proportion of pattern (I) in females and males, respectively. The majority of the residents (62.8% overall) have never been screened (pattern (IV)).

Results of the regression analyses are shown in Table 6. After adjustment, the cohort aged ≥81 years were statistically significantly less likely to adopt any of participation patterns (I–III) than the cohort aged 21–30 years with OR ranging from 0.22 to 0.45, depending on the participation pattern. The other age cohorts were more likely to adopt participation pattern (I–III) than the cohort of 21–30 years, except for the cohort of 71–80 years that had OR less than 1.0 in pattern (II). Female residents were significantly more likely to participate in the WBC monitoring in any participation pattern (I–III) than males, with OR ranging from 1.41 to 1.56.

In addition, original residential areas at the time of the incident were significantly associated with WBC participation behavior, with those who lived in the Evacuation Order Zone and Emergency Evacuation-Ready Zone more likely to adopt pattern (I–III) than those who lived outside any evacuation zones. In comparison with those who did not evacuate after the incident (non-evacuation), those who evacuated but did not return (history (a)) were significantly less likely to adopt participation pattern (I–III). In addition, those with evacuation history (b) were more likely to adopt the participation pattern (III) than non-evacuees with OR of 1.18 (95% CI: 1.08–1.29). We also observed a significant positive correlation of the participation behavior with the air dose rate (*p* < 0.001), which may indicate that those living in higher air contamination levels were more likely to take part in some monitoring (i.e., adopt pattern (I–III)).

As a sensitivity analysis, we modified the model using a different definition of ‘evacuation’—a movement away from the original residential area beyond *Chiku* instead of *Ku*; and obtained the similar results (see Table S1). In addition, we considered an interaction term between ‘original residential area’ and ‘evacuation history’ in order to see if the effect of evacuation history on the monitoring participation pattern differed by original residential area. However, we did not detect any statistical significance of this interaction term, so excluded it from the model.

Results of the sub-analysis for the consideration of ‘being detected’ with Cs are shown in Table S2. The detection rate of Cs in 2011–2012 was 23.3% (n = 3209 out of 13,750 individuals who participated in the WBC monitoring after September 2011 up to 31 March 2013 using FASTSCAN at MMGH or WBC-R43-22458 at Watanabe Hospital). The multiple regression analysis demonstrated that detection of Cs was not significantly associated with participation behavior.

## 4. Discussion

We assessed the voluntary WBC monitoring prevalence in adult cohorts (ages ≥21 years) in Minamisoma City, Fukushima Prefecture, in the first two years (2011–2012) and subsequent two years (2013–2014) following the 2011 Fukushima nuclear incident, and estimated the prevalence change between these time periods. We also evaluated the factors associated with monitoring prevalence, and characteristics of participants that influenced monitoring prevalence. We observed a low monitoring prevalence (30.2%) in 2011–2012 which decreased to 17.9% in 2013–2014 (Table 2). A potential explanation for the observed decrease in monitoring participation over time is that public concern about internal exposure risk might have decreased as several national and international bodies have acknowledged that the contribution to internal dose from internal exposure is small [9,11,28,29]. Also, it seems likely that once people have confirmed their internal contamination levels are low, they would be less likely to repeat the internal contamination measurements. 

We also found that the monitoring prevalence and behaviors were significantly associated with age, gender, pre-incident and post-incident dwelling area, evacuation history, and air dose rate at the post-incident dwelling area. Based on the discussions from many recent studies on health-related monitoring participation behavior [30,31,32,33,34,35], we elaborated the implications from the findings of this study around the concepts of ‘radiation risk perception’, defined as a cognitive process through which individuals perceive potential radiation risks, which determines their behavioral response to information or warnings on radiation; and ‘accessibility to the monitoring’ in terms of transportation and work schedules. Note that our intention in the present study is not to question the default setting of the monitoring program (mandatory or voluntary) or the low monitoring prevalence, nor to simply encourage monitoring participation, but to provide discussion points regarding in what manner internal contamination monitoring programs following a major radiation-release incident should be designed and delivered from a long-term viewpoint, i.e. during the recovery phase of the incident.

### 4.1. Radiation Risk Perception 

In general females are more likely than males to participate in health-related research studies/surveys, etc. [36,37,38], and it is known that females have higher health risk perception than males [39]. In the present study, we found that female residents had higher monitoring prevalence than males after adjustment for covariates in both 2011–2012 and 2013–2014 (Table 3). According to other studies after the Fukushima incident, females have higher radiation risk perception than males, i.e., worry more about potential adverse health effects of radiation exposure post-incident [40,41,42]. Thus, it is not surprising that those who have higher risk perception are more likely to volunteer for internal contamination monitoring. 

Our regression estimates also demonstrated that those who were living in the Evacuation Order Zone at the time of the incident (and therefore forced to evacuate) were more likely to attend internal monitoring than those living outside the evacuation zones (Table 3 and Table 6). These results also indicate the potential powerful link between the degree (magnitude) of radiation risk perception among affected people and the evacuation instructions they received. Suzuki et al. [40] and Hino et al. [41] also suggested in their post-Fukushima incident studies that having a history of evacuation correlated to a higher risk perception of radiation.

In addition, those who were living in the Evacuation Order Zone or Emergency Evacuation-Ready Zone at the time of the incident and then living inside Minamisoma City after the incident, were also more likely to have the internal monitoring than those living outside Minamisoma City after the incident (Table 4). Living in areas with higher air dose rate also resulted in higher odds of participating in an internal monitoring (Table 6). These findings also imply a relationship between radiation risk perception and living location relative to the nuclear plant at the time of or after the incident, or levels of radiation exposure. Here note that the internal contamination is marginal among most of the residents in the affected areas [9,11]. In Minamisoma City, the recent detection rate of Cs (during 1 April to 30 September 2015) among those aged 16 and over was only 0.6% [43]. It is also acknowledged by Tsubokura et al. that levels of soil contamination—a major indicator of air dose rate—were not necessarily associated with levels of internal contamination after the Fukushima incident [5], so we stress that radiation risk perception does not always correspond to actual risk.

In the context of risk perception, it may be reasonable to think that those who were detected with internal contamination in their initial WBC measurement may increase their perceived risk, and so would be more likely to take part in a subsequent monitoring than those in whom internal contamination was not detected. However, our study showed that there was no significant relationship between the detection of Cs and subsequent participation behavior (Table S2). This may be because although Cs was detected, participants were reassured by medical professions that the level of contamination was not so high as to increase future cancer risk [9,44], which might result in reduced anxiety and thus in decreased interest in the continued monitoring of their internal contamination. 

#### 4.1.1. Internal Contamination Monitoring as a Risk Communication Tool

Our finding of no observable change in monitoring behavior following a Cs detected result also hints at the possibility that internal contamination monitoring can work not only for informing public bodies about internal contamination levels, but also as a risk communication tool, alleviating individuals' concern and anxiety over radiation contamination. For example, in Minamisoma City, monitoring participants with internal ^137^ Cs contamination of more than 50 Bq/kg at the time of their first measurement/participation are offered counseling from medical professions about risky food intake, and were advised to consume mainly distributed food and to refrain from consuming potentially highly contaminated unmonitored foods, such as outdoor-grown mushrooms, mountain vegetables, and game meat (such as deer and wild boar, which are popular game foods in northern Japan, of which Fukushima Prefecture is a part) [45]. Thus, the findings of this study—on the factors associated with the monitoring participation behavior—should help enhance the discussion around what sort of individuals the internal contamination monitoring can or should approach as a risk communication tool, to alleviate anxiety, but also support risk reduction measures. Note that at the end of 2013 the world’s first WBC dedicated to babies and small children, called the BABYSCAN was developed and installed at MMGH to fulfill the requests of parents in Minamisoma City, offering both monitoring and individual counseling to parents of babies [46]. It is known that anxiety about radiation risks was associated with psychological distress [40]: mental health among adult forced-evacuees has been also recognized as the most serious, persisting health issue after the Fukushima incident [47].

### 4.2. Accessibility to the Monitoring 

We observed that those evacuated by 1 June 2011, but returned to the original residential area by 1 June 2013 were more likely than non-evacuees to participate in the monitoring only in 2013–2014 (Table 6). These individuals may have wanted to participate in the WBC monitoring soon after the incident, but were not able to do so because of evacuation; then, once they returned, participation became possible. This suggests that in addition to radiation risk perception, ease of access to monitoring and/or opportunity to participate may exert an influence on monitoring participation behavior. This also implies that the monitoring program can also work for returnees as a communication tool for risk management.

This concept of ‘accessibility to the monitoring’ could also explain the higher female monitoring prevalence than male. In Japan, there are substantially more female than male employees working part-time or unemployed [48]. Part-time and unemployed persons may find the monitoring program more accessible (in terms of time) during its working hours (basically Monday–Friday from 09:00 to 17:00, except for weekends and holidays), resulting in a higher monitoring prevalence in females than males. Similarly, the relatively low monitoring prevalence in the working-age population (the cohorts of 51–60 years or younger) (Table 2) could be partially explained by their difficultly in accommodating the monitoring during their work schedules.

### 4.3. Late-Stage Elderly Cohorts

Previous studies have demonstrated that elderly people have higher radiation risk perception than young individuals, and are more concerned about radiation exposure risk post-incident [40,41]. In our study, in fact, there was a clear tendency in the RP of the monitoring in 2013–2014 vs. 2011–2012 that higher the age, higher the RP; so that although all ages were less likely to attend monitoring in 2013–2014, the elderly cohort was more likely to participate in subsequent monitoring than younger individuals (Table 2). However, the age cohort with the lowest monitoring prevalence in 2011–2012 was that of ≥81 years cohort for both females and males (9.4% and 13.6% respectively), and in 2013–2014 females in this cohort retained this lowest monitoring prevalence, at 6.2% (Table 2). It may be the case that although some people in this late-stage elderly cohort have a strong desire to participate in monitoring, limited transportation or mobility due to health conditions hinder their access to the WBC-installed hospitals. 

Another possible, but important explanation may be that while it is known that in general elderly people have higher radiation risk perception than young individuals post-Fukushima incident [40,41], in the present study the later-stage elderly cohort might have potentially lower radiation risk perception than early-stage elderly cohorts and other younger cohorts, based on balancing the potential health burden due to Fukushima incident-attributable cancer in some years later and health benefits attainable through the monitoring; in other words, the later-stage elderly less concerned about their future cancer risk, so are less likely to participate in the WBC monitoring.

It should also be noted that there is a possibility that among the later-stage elderly cohort, the decision to participate in the WBC monitoring was highly influenced by their family members' or friends' opinions. This family/friend influence is evident among studies of cancer patients, where elderly people may prefer to receive less information about their health from medical professions, and be reluctant to participate in their health-related decision-making; instead, they may rely on information and decision by family members or friends who are more likely to address their emotional needs [49]. Given the above, and without further qualitative data, it is impossible to say whether the monitoring participation behavior observed in the late-stage elderly cohort reflects a true effect of individual attributes estimated in the analyses above. 

### 4.4. Future Recommendations

Given the findings of this study, we highlight the following points regarding the voluntary-based internal radiation monitoring program in Minamisoma City, and their relevance for a future radiation-release incident:

(1) Accessibility to the monitoring program (in terms of distance and operating times) may influence the monitoring participation behavior—there may exist some people who want to participate in the monitoring, but are not able to do so due to reasons beyond their personal control (e.g., difficult work schedules, limited transportation, mobility due to health conditions, etc.), indicating a gap in the monitoring delivery between the supply-side (monitoring providers) and demand-side (participants). In order to minimize this gap, the monitoring providers should consider, as one solution, the use of a mobile WBC unit (i.e., a WBC unit installed in a bus-like trailer for easy transportation), which can move around the city, and operate over a wider range of hours, to reduce the access barrier for these participants.
(2) Radiation risk perception may influence monitoring participation behavior, and thus people who are concerned about radiation exposure might be more likely to participate in voluntary-based monitoring. Since radiation risk perception also associates with dietary consumption behavior [50], monitoring results based on voluntary participation may not represent the exposure levels of the general population. Policymakers and implementers as well as researchers should carefully consider the potential for and effects of this bias. Note that because the internal contamination level has been very limited in Minamisoma City [9,12], this bias is likely to be minimal.
(3) The function of internal contamination monitoring should not be considered simply as informing personal and public health measures to ensure internal contamination levels are sufficiently low. Monitoring may also function as a risk communication tool, alleviating individuals’ concern and anxiety over radiation contamination and enhancing their well-being; however, this remains conjecture. Rigorous investigation is, therefore, needed to evaluate if, how much, and in what way the internal contamination monitoring can work so.

### 4.5. Limitations

This study has several limitations. First of all, the WBC monitoring program considered in this study was only offered to the Minamsoma City residents, potentially biasing the results, and limiting our ability to generalize to the wider population beyond the city. Second, while our study observation period was split at March 2013 (i.e., 1 July, 2011 to 31 March 2013; and 1 April 2013 to 31 March 2015), denominator populations of the WBC monitoring prevalence for each study observation period were based on the total number of registered residents at 1 June 2011 and 1 June 2013, respectively, because of data availability in the evacuation database (and Basic Resident Register). This means that there are some time gaps between the observation periods and time point of the evacuation/residential data considered, potentially biasing the monitoring prevalence. Third, we were not able to consider any uncertainty about the evacuation database (i.e., dwelling address was self-reported data). Fourth, no qualitative data was available to understand why people participate in the monitoring, i.e., what are the motivators and deterrents to participation; so we are not able to validate our discussion around radiation risk perception and accessibility to the monitoring. Finally, it should be noted that there are increasing numbers of nation-wide population cancer screening studies that also address the issue of low screening prevalence, which appear to be decreasing with time. Recent studies have focused mainly on the identification of factors associated with the screening prevalence [32]—the main interest in this study—and the evaluation of the cost-effective approaches to increase the screening prevalence [33,34,35]. However, given the substantially different context in which a screening is provided (e.g., human, material, and financial resources for operation and maintenance for providers; and financial assistance or special insurance and accessibility for participants), we were not easily able to compare our findings with those from these cancer studies.

## 5. Conclusions 

This is the first study to evaluate the voluntary internal radiation contamination monitoring prevalence and monitoring participation behavior after Japan’s 2011 Fukushima nuclear incident. The monitoring prevalence in 2011–2012 was only 30.2%, and this decreased to 17.9% in 2013–2014. Our intention in the present study is not to question the default setting of the monitoring (mandatory or voluntary) or the low monitoring prevalence or to encourage monitoring participation, but to feed into discussions on the concepts of 'radiation risk perception' and 'access to monitoring', and support societal decision-making on the continued operation of the Minamisoma monitoring program under the current low monitoring prevalence; and inform the global planning and preparedness of the internal contamination monitoring programs for similar crisis in the future. 

## Figures and Tables

**Table 1:** Pre-incident and post-incident demographic characteristics of all residents (number, %).

Characteristics	Number (%)
**Age at 11 March 2011**	
21–30	4012 (8.8)
31–40	6692 (14.6)
41–50	6663 (14.6)
51–60	9550 (20.9)
61–70	9113 (19.9)
71–80	6459 (14.1)
≥81	3299 (7.2)
**Gender**	
Male	21929 (47.9)
Female	23859 (52.1)
**Original residential area by evacuation instruction**	
Outside the evacuation zones	6722 (14.7)
Evacuation Order Zone	9471 (20.7)
Emergency Evacuation-Ready Zone	29595 (64.6)
**Post-incident dwelling area at 1 June 2011**	
Inside Minamisoma City	24926 (51.2)
Inside Fukushima/Outside Minamisoma City	7875 (16.2)
Neighboring Prefectures	7589 (15.6)
Outside Fukushima & neighboring Prefectures	5398 (11.1)
**Post-incident dwelling area at 1 June 2013**	
Inside Minamisoma City	36074 (74.2)
Inside Fukushima/Outside Minamisoma City	4107 (8.4)
Neighboring Prefectures	3437 (7.1)
Outside Fukushima & neighboring Prefectures	2170 (4.5)

**Table 2:** While body counter examination prevalence (number) and relative prevalence (RP) for 2013–2014 vs. 2011–2012.

	2011–2012	2013–2014	RP (95% Confidence Interval)
**Male**			
**Age at 11 March 2011**			
21–30	22.1 (481)	7.2 (120)	0.33 (0.27–0.40) ***
31–40	28.6 (983)	11.1 (362)	0.39 (0.35–0.43) ***
41–50	28.0 (940)	11.5 (384)	0.41 (0.37–0.46) ***
51–60	26.4 (1253)	14.2 (604)	0.54 (0.49–0.59) ***
61–70	31.7 (1433)	22.4 (1110)	0.71 (0.66–0.75) ***
71–80	28.0 (747)	24.5 (737)	0.88 (0.80–0.96) **
≥81	13.6 (138)	12.6 (181)	0.93 (0.75–1.13)
Total	27.2 (5975)	16.0 (3498)	0.59 (0.56–0.61) ***
**Female**			
**Age at 11 March 2011**			
21–30	35.9 (658)	19.3 (264)	0.54 (0.47–0.61) ***
31–40	41.2 (1341)	17.8 (540)	0.43 (0.40–0.47) ***
41–50	37.5 (1239)	18.0 (582)	0.48 (0.44–0.52) ***
51–60	36.8 (1767)	22.6 (987)	0.62 (0.58–0.66) ***
61–70	36.9 (1692)	27.4 (1356)	0.74 (0.70–0.79) ***
71–80	24.6 (931)	19.7 (767)	0.80 (0.74–0.87) ***
≥81	9.4 (215)	6.2 (187)	0.66 (0.55–0.80) ***
Total	32.9 (7843)	19.6 (4683)	0.60 (0.58–0.62) ***
Overall	30.2 (13818)	17.9 (8181)	0.59 (0.58–0.61) ***

Note: ** *p* < 0.01; *** *p* < 0.001.

**Table 3:** Negative binomial regression model for the factors associated with the while body counter monitoring prevalence in 2011–2012 and 2013–2014 (PR, 95% CI).

	2011–2012	2013–2014
**Age at 11 March 2011**		
21–30	1.00	1.00
31–40	1.21 (1.09–1.35) ***	1.14 (0.92–1.40)
41–50	1.10 (0.99–1.22)	1.12 (0.91–1.38)
51–60	1.06 (0.96–1.18)	1.47 (1.19–1.81) ***
61–70	1.15 (1.04–1.28) **	1.80 (1.47–2.21) ***
71–80	0.86 (0.77–0.96) **	1.64 (1.32–2.03) ***
≥81	0.35 (0.30–0.41) ***	0.59 (0.46–0.75) ***
**Gender**		
Male	1.00	1.00
Female	1.25 (1.18–1.32) ***	1.32 (1.19–1.47) ***
**Original residential area by evacuation instruction**		
Outside the evacuation zones	1.00	1.00
Evacuation Order Zone	1.78 (1.57–2.03) ***	1.45 (1.20–1.73) ***
Emergency Evacuation-Ready Zone	1.51 (1.36–1.68) ***	1.31 (1.10–1.56) **
Post-incident dwelling area		
Inside Minamisoma City	1.00	1.00
Inside Fukushima/Outside Minamisoma City	0.99 (0.81–1.21)	1.05 (0.68–1.61)
Neighboring Prefectures	0.97 (0.83–1.20)	0.64 (0.38–1.09)
Outside Fukushima & neighboring Prefectures	0.92 (0.71–1.19)	0.33 (0.14–0.81) *

Note: PR = prevalence ratio; CI = confidence interval; * *p* < 0.05; ** *p* < 0.01; *** *p* < 0.001.

**Table 4:** Effect of post-incident dwelling area between on while body counter monitoring prevalence by original residential area in 2011–2012 and 2013–2014 (PR, 95% CI).

Original Residential Area by Evacuation Instruction	Post-Incident Dwelling Area	2011–2012	2013–2014
Outside the evacuation zones	Inside Minamisoma City	1.00	1.00
	Inside Fukushima/Outside Minamisoma City	0.99 (0.81–1.21)	1.05 (0.68–1.61)
	Neighboring Prefectures	1.00 (0.83–1.20)	0.64 (0.38–1.09)
	Outside Fukushima & neighboring Prefectures	0.92 (0.71–1.19)	0.33 (0.14–0.81) *
Evacuation Order Zone	Inside Minamisoma City	1.00	1.00
	Inside Fukushima/Outside Minamisoma City	0.84 (0.74–0.95) **	0.66 (0.54–0.82) ***
	Neighboring Prefectures	0.64 (0.56–0.73) ***	0.29 (0.22–0.39) ***
	Outside Fukushima & neighboring Prefectures	0.48 (0.41–0.56) ***	0.12 (0.08–0.19) ***
Emergency Evacuation-Ready Zone	Inside Minamisoma City	1.00	1.00
	Inside Fukushima/Outside Minamisoma City	0.98 (0.88–1.09)	0.84 (0.69–1.02)
	Neighboring Prefectures	0.78 (0.70–0.87) ***	0.56 (0.45–0.69) ***
	Outside Fukushima & neighboring Prefectures	0.69 (0.61–0.77) ***	0.45 (0.35–0.58) ***

Note: PR = prevalence ratio; CI = confidence interval; * *p* < 0.05; ** *p* < 0.01; *** *p* < 0.001 across columns. This is an interaction term of ‘original residential area’ and ‘post-incident dwelling area’ in the model presented in Table 3.

**Table 5:** While body counter monitoring participation patterns (%, number).

	Pattern (I)	Pattern (II)	Pattern (III)	Pattern (IV) (non-participation)
**Male**				
**Age at 11 March 2011**				
21–30	3.8 (83)	18.2 (398)	3.5 (76)	74.5 (1624)
31–40	7.1 (244)	21.5 (739)	4.7 (161)	66.7 (2294)
41–50	6.7 (225)	21.3 (715)	4.9 (163)	67.1 (2253)
51–60	7.9 (377)	18.5 (876)	7.9 (375)	65.7 (3118)
61–70	12.9 (583)	18.8 (850)	10.7 (484)	57.6 (2606)
71–80	11.5 (307)	16.5 (440)	11.3 (301)	60.8 (1624)
≥81	5.1 (52)	8.5 (86)	6.6 (67)	79.8 (808)
Total	8.5 (1871)	18.7 (4104)	7.4 (1627)	65.3 (14327)
**Female**				
**Age at 11 March 2011**				
21–30	10.6 (194)	25.3 (464)	8.1 (148)	56.0 (1025)
31–40	10.6 (344)	30.6 (997)	7.3 (237)	51.5 (1676)
41–50	10.7 (353)	26.8 (886)	7.3 (241)	55.2 (1827)
51–60	12.9 (619)	23.9 (1148)	11.7 (562)	51.5 (2475)
61–70	15.0 (690)	21.8 (1002)	11.9 (545)	51.3 (2353)
71–80	8.4 (319)	16.2 (612)	8.5 (321)	66.9 (2535)
≥81	2.3 (53)	7.1 (162)	2.5 (57)	88.1 (2014)
Total	10.8 (2572)	22.1 (5271)	8.8 (2111)	58.3 (13,905)
Overall	9.7 (4443)	20.5 (9375)	8.2 (3738)	61.7 (28,232)

Note: Participation pattern (I) participated both in 2011–2012 and 2013–2014; (II) participated only in 2011–2012; (III) participated only in 2013–2014; and (IV) participated neither in 2011–2012 nor 2013–2014 (non-participation).

**Table 6:** Multinomial logistic regression model (reference group: non-participation) for the factors associated with the whole body counter monitoring participation behavior (OR, 95% CI).

	Pattern (I)	Pattern (II)	Pattern (III)
**Age at 11 March 2011**			
21–30	1.00	1.00	1.00
31–40	1.41 (1.21–1.64) ***	1.34 (1.22–1.48) ***	1.18 (0.99–1.40)
41–50	1.28 (1.10–1.49) **	1.16 (1.05–1.28) **	1.15 (0.97–1.36)
51–60	1.55 (1.34–1.79) ***	1.04 (0.94–1.14)	1.91 (1.64–2.23) ***
61–70	2.25 (1.95–2.58) ***	1.08 (0.99–1.19)	2.35 (2.02–2.74) ***
71–80	1.24 (1.07–1.44) **	0.70 (0.63–0.78) ***	1.63 (1.39–1.92) ***
≥81	0.28 (0.23–0.36) ***	0.22 (0.19–0.26) ***	0.45 (0.36–0.57) ***
**Gender**			
Male	1.00	1.00	1.00
Female	1.56 (1.46–1.66) ***	1.48 (1.41–1.55) ***	1.41 (1.32–1.52) ***
**Original residential area by evacuation instruction**			
Outside the evacuation zones	1.00	1.00	1.00
Evacuation Order Zone	3.17 (2.70–3.71) ***	2.03 (1.82–2.27) ***	1.27 (1.09–1.48) **
Emergency Evacuation-Ready Zone	1.88 (1.69–1.09) ***	1.47 (1.36–1.58) ***	1.03 (0.93–1.14)
Evacuation history (beyond *Ku*)			
History (a)	0.45 (0.40–0.51) ***	0.58 (0.54–0.63) ***	0.74 (0.65–0.83) ***
History (b)	1.06 (0.97–1.15)	0.96 (0.90–1.03)	1.18 (1.08–1.29) ***
History (c)	0.89 (0.74–1.07)	1.00 (0.88–1.14)	1.17 (0.96–1.43)
History (d) (non-evacuation)	1.00	1.00	1.00
Air dose rate [μSv/h] as of 22 April 2011 at original residential area	1.17 (1.15–1.19) ***	1.21 (1.19–1.23) ***	1.07 (1.04–1.10) ***

Note: ** *p* < 0.01; *** *p* < 0.001. across columns. Participation pattern (I) participated both in 2011–2012 and 2013–2014; (II) participated only in 2011–2012; (III) participated only in 2013–2014; and (IV) participated neither in 2011–2012 nor 2013–2014 (non-participation). Evacuation history (a) evacuated both in 2011–2012 and 2013–2014; (b) evacuated in 2011–2012, but returned to original residential area by 2013–2014; (c) evacuated only during 2013–2014; and (d) evacuated in neither 2011–2012 nor 2013–2014 (non-evacuation).

## Supplementary Materials

Table S1: Multinomial logistic regression model (reference group: non-monitoring) for the factors associated with the WBC monitoring participation behavior (OR, 95% CI), Table S2: Logistic regression model for the effect of Cs-detection on having the subsequent monitoring participation (OR, 95% CI).
