# Supplementary material for: Towards a Long-Term Strategy for Voluntary-Based Internal Radiation Contamination Monitoring: A Population-Level Analysis of Monitoring Prevalence and Factors Associated with Monitoring Participation Behavior in Fukushima, Japan
Source: Int J Environ Res Public Health. 2017 Apr 9;14(4):397. doi: 10.3390/ijerph14040397 (PMC5409598; doi:10.3390/ijerph14040397)
Supplement: Supplementary file 1 [file ijerph-14-00397-s001.pdf]

Table S1. Multinomial logistic regression model (reference group: non-monitoring) for the factors associated with the WBC monitoring participation behavior (OR, 95% CI). * p<0.05, ** p<0.01, *** p<0.001 across columns. Participation pattern (I) participated both in 2011–2012 and 2013–2014; (II) participated only in 2011–2012; (III) participated only in 2013–2014; and (IV) participated neither in 2011–2012 nor 2013–2014 (non-participation). Evacuation history (a) evacuated both in 2011–2012 and 2013–2014; (b) evacuated in 2011–2012, but returned to original residential area by 2013–2014; (c) evacuated only during 2013–2014; and (d) evacuated in neither 2011–2012 nor 2013–2014 (non-evacuation).

	Pattern (I)	Pattern (II)	Pattern (III)
Age at March 11, 2011			
21–30	1.00	1.00	1.00
31–40	1.41 (1.21–1.64)***	1.34 (1.22–1.48)***	1.18 (1.00–1.40)
41–50	1.28 (1.10–1.49)**	1.16 (1.05–1.28)**	1.16 (0.98–1.38)
51–60	1.56 (1.36–1.80)***	1.05 (0.95–1.15)	1.95 (1.67–2.28)***
61–70	2.26 (1.96–2.59)***	1.09 (0.99–1.20)	2.40 (2.06–2.80)***
71–80	1.25 (1.07–1.45)**	0.71 (0.63–0.78)***	1.67 (1.42–1.96)***
81–	0.29 (0.23–0.36)***	0.23 (0.20–0.27)***	0.47 (0.37–0.59)***
Gender			
Male	1.00	1.00	1.00
Female	1.55 (1.45–1.65)***	1.47 (1.40–1.54)***	1.40 (1.31–1.51)***
Original residential area			
Outside the evacuation zones	1.00	1.00	1.00
Evacuation Order Zone	2.81 (2.40–3.30)***	1.86 (1.67–2.07)***	1.07 (0.93–1.25)
Emergency Evacuation-Ready Zone	1.83 (1.65–2.04)***	1.44 (1.34–1.55)***	1.01 (0.92–1.11)
Evacuation history (beyond *Chiku*)			
History (a)	0.53 (0.47–0.59)***	0.65 (0.60–0.70)***	0.90 (0.80–1.00)
History (b)	1.11 (1.02–1.21)*	0.99 (0.92–1.06)	1.24 (1.13–1.37)***
History (c)	0.90 (0.75–1.08)	1.04 (0.91–1.17)	1.21 (1.00–1.46)
History (d) (non-evacuation)	1.00	1.00	1.00
Air dose rate [μSv/h] as of April 22, 2011 at original residential area	1.16 (1.14–1.19)***	1.20 (1.18–1.22)***	1.07 (1.04–1.09)***

Table S2. Logistic regression model for the effect of Cs-detection on having the subsequent monitoring participation (OR, 95% CI). * p<0.05, ** p<0.01, *** p<0.001. Evacuation history (a) evacuated both in 2011–2012 and 2013–2014; (b) evacuated in 2011–2012, but returned to original residential area by 2013–2014; (c) evacuated only during 2013–2014; and (d) evacuated in neither 2011–2012 nor 2013–2014 (non-evacuation).

Detection	
No	1.00
Yes	0.96 (0.88–1.06)
Age at March 11, 2011	
21–30	1.00
31–40	1.06 (0.90–1.25)
41–50	1.11 (0.94–1.31)
51–60	1.49 (1.28–1.75)***
61–70	2.07 (1.78–2.42)***
71–80	1.77 (1.50–2.10)***
81–	1.26 (0.97–1.65)
Gender	
Male	1.00
Female	1.07 (1.00–1.16)
Original residential area	
Outside the evacuation zones	0.66 (0.55–0.79)***
Evacuation Order Zone	
Planned Evacuation Zone	0.87 (0.08–9.69)
Emergency Evacuation-Ready Zone	0.81 (0.70–0.93)**
Evacuation history	
History (a)	0.79 (0.69–0.91)**
History (b)	1.09 (0.99–1.20)
History (c)	0.90 (0.74–1.10)
History (d) (non-evacuation)	1.00
Air dose rate [μSv/h] as of April 22, 2011 at original residential area	0.97 (0.95–0.99)**
